# Vertigo Associated With Cochlear Implant Surgery: Correlation With Vertigo Diagnostic Result, Electrode Carrier, and Insertion Angle

**DOI:** 10.3389/fneur.2021.663386

**Published:** 2021-06-11

**Authors:** Charlotte Weinmann, Uwe Baumann, Martin Leinung, Timo Stöver, Silke Helbig

**Affiliations:** ^1^Department of Otorhinolaryngology, Goethe-University Frankfurt, Frankfurt, Germany; ^2^Department of Audiological Acoustics, Goethe-University Frankfurt, Frankfurt, Germany

**Keywords:** cochlear implant, vestibular function, questionnaire, electrode design, vertigo

## Abstract

**Objective:** Vertigo is a common side effect of cochlear implant (CI) treatment. This prospective study examines the incidence of postoperative vertigo over time and aims to analyze influencing factors such as electrode design and insertion angle (IA).

**Study Design and Setting:** This is a prospective study which has been conducted at a tertiary referral center (academic hospital).

**Patients:** A total of 29 adults were enrolled and received a unilateral CI using one of six different electrode carriers, which were categorized into “structure-preserving” (I), “potentially structure-preserving” (II), and “not structure-preserving” (III).

**Intervention:** Subjective vertigo was assessed by questionnaires at five different time-points before up to 6 months after surgery. The participants were divided into four groups depending on the time of the presence of vertigo before and after surgery. Preoperatively and at 6 months postoperatively, a comprehensive vertigo diagnosis consisting of Romberg test, Unterberger test, subjective visual vertical, optokinetic test, video head impulse test, and caloric irrigation test was performed. In addition, the IA was determined, and the patients were divided in two groups (<430°; ≥430°).

**Main Outcome Measures:** The incidence of vertigo after CI surgery (group 1) was reported, as well as the correlation of subjective vertigo with electrode array categories (I–III) and IA.

**Results:** Among the participants, 45.8% experienced new vertigo after implantation. Based on the questionnaire data, a vestibular origin was suspected in 72.7%. The results did not show a significant correlation with subjective vertigo for any of the performed tests. In group 1 with postoperative vertigo, 18% of patients showed conspicuous results in a quantitative analysis of caloric irrigation test despite the fact that the category I or II electrodes were implanted, which are suitable for structure preservation. Average IA was 404° for the overall group and 409° for group 1. There was no statistically significant correlation between IA and perceived vertigo.

**Conclusions:** Though vertigo after CI surgery seems to be a common complication, the test battery used here could not objectify the symptoms. Further studies should clarify whether this is due to the multifactorial cause of vertigo or to the lack of sensitivity of the tests currently in use. The proof of reduced probability for vertigo when using atraumatic electrode carrier was not successful, nor was the proof of a negative influence of the insertion depth.

## Introduction

Since the beginning of cochlear implant (CI) surgery, vertigo with vestibular origin is known as a typical postoperative side effect and has been described by various authors (1–4). So far, little is known about the factors that increase the risk of vertigo. Within the last decades, the indication criteria for a CI have expanded, and therefore the number of patients who received this neuro-prosthesis increased. Today patients do not have to suffer from complete deafness, and also candidates with low-frequency residual hearing and unilateral hearing loss are eligible for this treatment (5). Surgery in terms of hearing preservation appears to reduce the postoperative risk of vertigo (6). While age is discussed as a potential risk factor in several studies (7, 8), no significant correlation was found regarding gender and etiology of hearing loss (7, 9, 10).

In 2008, Todt et al. were able to show that the insertion of the electrode array through the round window caused less damage to the vestibular organ. Therefore, this approach seems to be the most advantageous and is still favored in “structure-preserving surgery” (11). In addition, electrode arrays were redesigned to be very thin and flexible for insertion without surgically enlarging the round window and therefore helping to maintain the fragile intracochlear structures during insertion. The design and the insertion angle of the electrode carriers seem to influence the occurrence of postoperative vertigo in adult patients (12). However, despite these efforts to preserve the structure of the cochlea, current literature remains to describe that CI surgery carries a likely risk of vertigo. A postoperative pathology of the lateral semicircular canal, measured by caloric testing, as well as a reduced saccular function measured by cVEMPs was demonstrated by different authors (13, 14). However, conspicuous findings of different tests do not always seem to correlate statistically significant with the onset of vertigo. In a review of Krause et al. (15) on vertigo after cochlear implantation, it became evident that only caloric and VEMP test showed significant negative effects. In addition, quality, onset, and duration of perceived vertigo seems to be described differently by patients (1). Therefore, the question remains as to whether all vertigo symptoms originate by vestibular problems and whether factors that increase the risk of postoperative vertigo after CI surgery can be identified. For this reason, this prospective study was initiated to clarify these key questions.

## Materials and Methods

### Subjects

The prospective study presented here was preapproved by the ethics committee at the university hospital Frankfurt/M (no. 524/15). The inclusion criteria were as follows: the patient had to be at least 18 years old, had never received a CI before, and was planned for unilateral implantation. The exclusion criteria were unwillingness to participate in the study, being a minor, and CI re-implantation. Between 2016 and 2018, 32 patients were enrolled; all of them signed an informed consent to participate in the study. Three patients dropped out of the follow-up: one patient could not be implanted due to ossification of the cochlea, and two patients did not show up for follow-up appointments. In total, data obtained from 29 patients was available for evaluation, among them 16 (55%) were women and 13 (45%) were men. The mean age was 58 years (median 57 years, standard deviation ± 12.5 years). The causes of hearing loss ranged from sudden deafness (*n* = 5), trauma (*n* = 1), apoplexy (*n* = 1), otitis media (*n* = 1), and Ménière's disease (*n* = 2) to congenital deafness due to infection or hypoxia (*n* = 4) and deafness due to unclear etiology (*n* = 15).

It was intended to include a higher number of study participants, but within the observation period of 1.5 years, this could not be achieved. The reasons therefore included a high number of second ear (bilateral) CI surgeries, minority, limiting language barriers, or unwillingness to accept the burdens of vertigo testing without the presence of symptoms.

### Surgery

The procedure during surgery is a crucial factor with impact on the delicate intracochlear structures (16). Surgery was performed by three different surgeons and, in all cases, in a standardized manner with a focus on structural preservation within the cochlea. First, mastoidectomy and posterior tympanotomy were performed. Then, a glucocorticoid (triamcinolone) was flushed into the middle ear and left there while drilling the implant bed. Afterwards, the round window was visualized and, if necessary, the overhang was removed with diamond burrs using a reduced rotation speed of 8,000 rpm. Now the implant was placed in its skull bed. After cleaning of the middle ear and punctual opening of the round window, slow insertion of the electrode array was performed. In cases where its diameter did not permit direct insertion, the round window had been extended anterior-laterally according to the required dimensions. After opening the cochlea, suction was avoided.

### Electrode Carriers

Patients were provided with different cochlear implants from the following three manufacturers: Cochlear GmbH & Co. KG (Sydney, Australia), MED-EL GmbH (Innsbruck, Austria), and Advanced Bionics AG (Stäfa, Switzerland). Among the electrode carriers used, there were both “structure-preserving” and “not structure-preserving” design forms. The electrodes for which studies have shown that they have a higher probability of residual hearing preservation were classified as “potentially structure-preserving.” An overview of the electrodes used in the study is shown in Table 1.

**Table 1:** Implanted devices and electrode designs (for details on the devices and manufacturers, see section “Materials and Methods”).

**Electrode** ** category**	**Classification**	**Device**	**Electrode** ** design**	**Number of** ** patients**
I	Structure-preserving	CI522	Straight	3 (10%)
I	Structure-preserving	FLEX24	Straight	2 (7%)
II	Potentially structure-preserving	CI532	Preformed perimodiolar	5 (17%)
II	Potentially structure-preserving	FLEX28	Straight	7 (24%)
III	Not structure-preserving	HiFocus Mid-Scala	Preformed perimodiolar	1 (4%)
III	Not structure-preserving	CI512	Preformed perimodiolar	11 (38%)
				*n* = 29 (100%)

Eleven (38%) of the patients received the Cochlear™ Nucleus® CI512 (Contour Advance) electrode. This preformed electrode with 22 contacts was designed with the intention of allowing a close position to the modiolus (17). However, several studies showed that, due to its shape, size, and insertion procedure, this electrode array has the potential for a scalar transition and that it does not allow reliable hearing preservation (18, 19). Therefore, it was classified as “not structure-preserving” in our study. Three patients (10%) received the Cochlear™ Nucleus® CI522 (Slim Straight), which also has 22 platinum electrode contacts (20). It was developed to reduce the frequency of scalar translocation and to improve the odds of hearing preservation (18, 21). Five patients (17%) received the Cochlear™ Nucleus® Profile Implant CI532 (Slim Modiolar Electrode), launched in 2016 and designed as a preformed electrode with 22 platinum contacts and a maximum diameter of 0.4 mm at the electrode tip and 0.5 mm basal, which meant a reduction of volume by 60% compared to previous electrodes. Besides helping to protect the delicate structures of the cochlea (22), the preformed design allows placement close to the modiolus (23). Since it has been proven to enable the preservation of hearing (24), it was classified as “potentially structure-preserving” in this study.

In 2006, MED-EL introduced the straight and flexible FLEX electrode series, all equipped with 12 platinum contacts and currently available in various lengths. In our study, MED-EL® FLEX24 electrode arrays with a length of 24 mm were implanted in two (7%) patients, and the MED-EL® FLEX28 electrodes with a length of 28 mm were implanted in seven (24%) patients. The maximum diameter of both is 0.8 mm at the base, which makes round window insertion possible in most cases (25). FLEX24, in particular, is used for the hearing-preserving surgery of CI candidates with low-frequency residual hearing and classified as “structure-preserving” in this study. Despite its use as an electrode for the standard treatment of deaf or profoundly hearing-impaired patients, FLEX28 also has the potential to preserve structure. Therefore, it was classified here as “potentially structure-preserving.”

One patient (4%) received the Advanced Bionics HiFocus™ Mid-Scala electrode carrier. This electrode array, launched in 2013, is 15 mm in length, preformed, and has 16 platinum contacts and a maximum diameter of 0.7 mm at the basal end. The insertion is performed with a stylet similar to the procedure for the Contour Advance electrode from Cochlear™. Therefore, the Mid-Scala electrode carrier was classified as “not structure-preserving” in this study.

The 29 study participants were provided with five electrode carriers of electrode group I, 12 of group II, and 12 of group III (see Table 1).

### Electrode Insertion Depth

**Figure 1:**
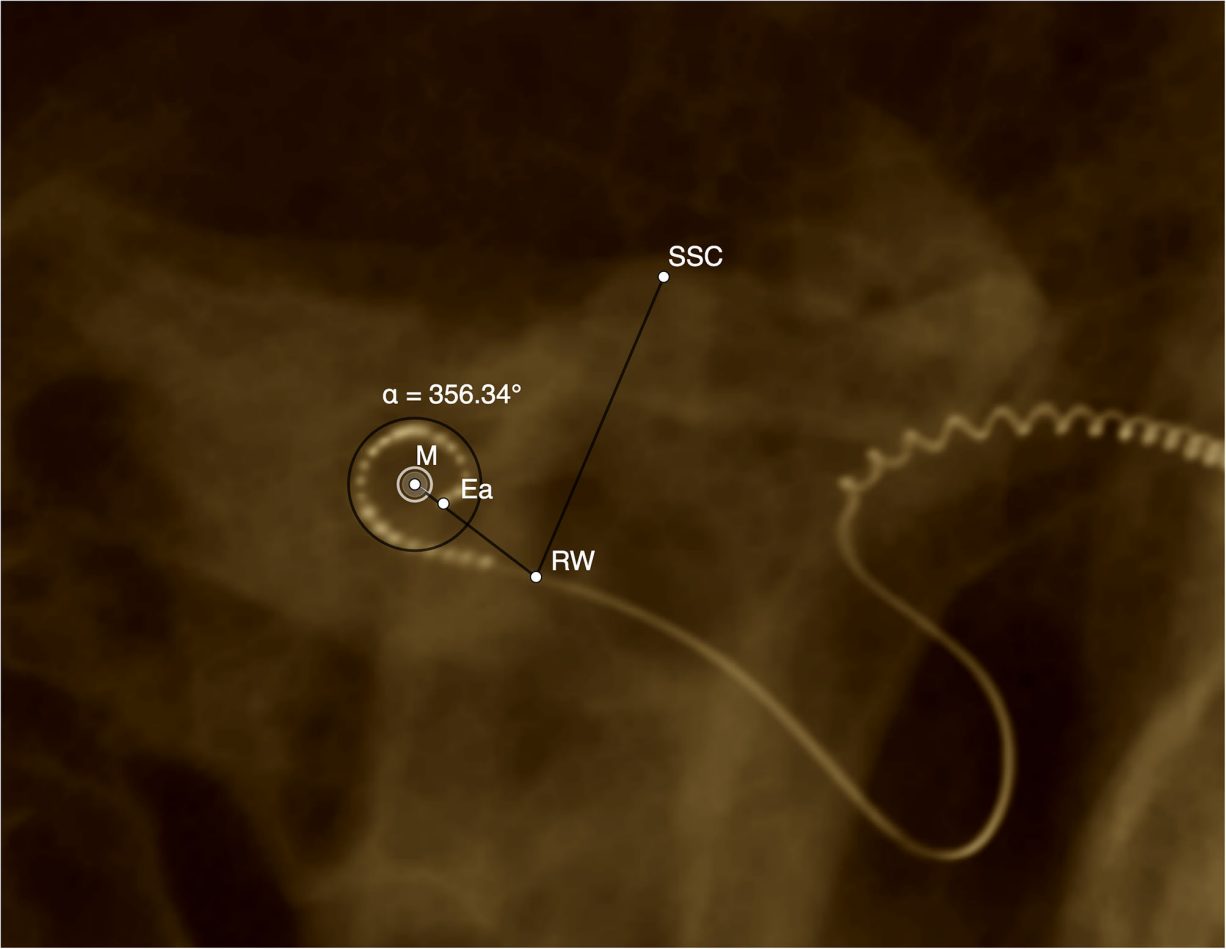
Example DVT reconstruction of CI512 device (left side, patient n20). Determination of insertion angle. M, modiolus; Ea, most apical electrode; RW, round window; SSC, superior semicircular canal; a, insertion angle (356.3°).

Insertion angle was measured based on postoperative CT images to verify the cochlear implant electrode position (26–28). Electrode insertion angle was determined manually by the application of GeoGebra geometrics software (Version Classic 6) (29) as shown in Figure 1. First, the upper semicircular canal (SSC) was identified on CT image. Next, the round window (RW) was marked by drawing a vertical line from the upper edge of the SSC through the semicircular channel (f). This vertical line crosses the electrode in the area of the round window, where the starting point for insertion angle determination is defined. The position of the modiolus (M) was determined, and the deepest apical electrode (Ea) was marked. In cases where the electrode was inserted at a maximum of 30°, the angle between M, RW, and Ea could be calculated directly. If a deeper insertion was present, a second circle was placed in the second turn of the cochlea, again determining the position of the modiolus (M2). The angle between M2, RW, and Ea was calculated, and this result was then summed with 360°.

To evaluate the influence of the insertion depth measured by the insertion angle, the patients were categorized into two groups. The decision to form two insertion angle groups and to select the subdivision at 430° was made after calculating the binomial distribution.

Angle category u included 20 patients, whose electrode was implanted with an insertion angle of <430°. Eight patients formed the angle category o, with an insertion angle greater than and equal to 430 degrees.

### Low-Frequency Residual Hearing

Pure tone audiograms of the 29 patients were tested preoperatively and postoperatively (mean = 5.5 M, minimum = 1 M, maximum = 14 M). The average of unaided air conduction thresholds for low frequencies at 125, 250, 500, and 1,000 Hz were calculated. The study participants without residual hearing before surgery (*n* = 2) were not considered for evaluation. Postoperative cases without residual hearing at maximum detectable levels, which were 85 dB HL at 125 Hz, 105 dB HL at 250 Hz, and 110 dB HL at 500 and 1,000 Hz, were also excluded. The rest were grouped by electrode category I to III, and statistical analysis was performed.

### Questionnaire

The subjective symptoms of vertigo in patients were assessed before and after implantation using five questionnaires published by Krause et al. (1) to evaluate the complaints of patients with vertigo. Other studies used the Dizziness Handicap Inventory (DHI) developed in 1990, which contains 25 questions that assess the relationship between vertigo symptoms and performance in everyday life (30, 31). The advantage of this standardized test is its reproducibility and comparability with other studies. However, it does not allow conclusions to be drawn about the cause of the vertigo, which is why we modified the test setting. The patients completed the questionnaires 1 day before surgery, 1 week after surgery, at the time of the initial adjustment of the processor, and 3 and 6 months after the initial adjustment. If vertigo was present in the first two questionnaires, detailed follow-up questions were asked, e.g., quality, frequency, duration. The degree of subjective impairment due to vertigo was indicated by the patients on a visual analog scale, with “0” representing “no impairment” and “10” representing “extreme impairment.” The last three questionnaires asked about the presence and change of vertigo symptoms. Based on the information obtained by the questionnaires on the occurrence of vertigo, four groups were formed:

Group 0: before and after surgery, no vertigo

Group 1: before surgery, no vertigo; after surgery, vertigo

Group 2: before surgery, vertigo; after surgery, no vertigo

Group 3: before and after surgery, vertigo

The patients were classified into three groups, based on the probable origin of their vestibular symptoms, using the criteria “quality of vertigo” and “accompanying symptoms”:

Group A: profound suspicion of vestibular origin (rotational vertigo, swaying vertigo or lifting sensation, *and* accompanying symptoms)

Group B: potential vestibular origin (rotational vertigo, swaying vertigo or lifting sensation *without* accompanying symptoms)

Group C: suspicion of central origin (general feeling of dysbalance).

### Vestibular Function Tests

Both preoperatively and at 6 months postoperatively, a comprehensive vertigo testing was conducted, consisting of Romberg test, Unterberger test, subjective visual vertical test (SVV), optokinetic test, video head impulse test (vHIT), and caloric irrigation test. Since only one investigator performed all the measurements within this study, investigator-dependent variation in the experimental procedure could be excluded. Preoperatively, all 29 patients participated in the Romberg test, Unterberger test, and the test of SVV. For the optokinetic test, preoperative data in four patients could not be used due to technical problems, leaving 25 preoperative data sets for evaluation. Due to the same problems, one data set was excluded postoperatively. The vHIT was refused by one patient preoperatively and one study participant postoperatively due to cervical pain, leaving 28 sets of data pre- and post-operatively for evaluation. The caloric irrigation test was performed in all 29 patients preoperatively. Postoperatively, two patients refused the test, and in one case, the irrigation device was unavailable, leaving the data sets of 26 subjects.

### Romberg Test

When performing the Romberg test, the patient was in an evenly lit and quiet room and stood upright on a firm surface. The patient stretched his arms forward, with his hands in a supine position. The test was initially carried out with the eyes open; if the patient was confident enough, the test was also carried out with the eyes closed. Swaying and falling tendencies in one direction were rated as conspicuous.

### Unterberger Test (Fukuda Test)

The Unterberger test was carried out in an evenly lit and quiet room. The patient stepped on the same spot 50 times with his eyes closed and arms extended forward. At each time, the thighs should be bent at a 90° angle (32). The direction of rotation of the patient was recorded. According to Biesinger and Iro (33), a deviation above 45° was considered conspicuous.

### Subjective Visual Vertical

A line was drawn centrally on the bottom inside a bucket. The examiner held the bucket horizontally in front of the patient's face so that the participant could look into it and see the line. The examiner turned the bucket 10 times in a row alternately to the left or right, and the patient should turn it back to the vertical using the line inside the bucket. The examiner then read off a possible deviation from the vertical using a plumb and a protractor, which were attached to the bucket. According to Böhmer (34), the SVV reflects lateral differences in the tonic affinity of the otolith organs (especially the utricle). In the presented study, a deviation of more than 2° was rated outside physiological range.

### Optokinetic Test

During the test, the patient was sitting on a fixed chair and wearing glasses with an integrated camera of the Visual Eyes 525 video oculography system (Interacoustics, Middelfart, Denmark). The participant looked at a wall in front where periodic, vertical, and yellow and blue stripe patterns were presented using a video projector. The patient was asked to observe a stripe in the center of the field and let the eyes follow the movement of the stripe until it disappeared. By then, the eyes jumped back to the center of the field, and the gaze was fixed on a new stripe. The speed of the stripes presented affected the slow phase velocity (SPV) of the eyes, which the stripes can normally follow up to a speed of 40°/s. The nystagmus movements were evaluated by the software system OtoAccessTM (Interacoustics, Middelfart, Denmark). The stimulus speeds used for the right and the left sides were 20, 35, and 50°/s, with a recording time of 20 s. The optokinetic nystagmus was assessed according to Haid et al. (35) as irregular if a nystagmus difference between the two eyes of greater than or equal to 20% (of the SPV) was measured to the right and to the left for the same speed. A conspicuous nystagmus difference occurs in patients with central vestibular damage, but not peripheral lesions (35, 36).

### Video Head Impulse Test

The vHIT, according to Halmagyi and Curthoys, is used to check the semicircular canals and the vestibulo-ocular reflex triggered by irritation as a reaction to stimuli in the high frequency range (37). In this study, the vHIT was used to check the integrity of the horizontal semicircular canals (hSCC). During the examination, the patient sat 1.5 m from a wall and fixed a point target at eye level while wearing “ICS® Impulse” glasses with an integrated camera (Natus Medical Incorporated, Pleasanton, USA), which recorded the eye movement of the right eye and passed it on to the OTOsuite® UNIVERSE audiometry (Natus Medical Incorporated, Pleasanton, USA). The examiner, standing behind the test person, alternately and as unpredictably as possible performed movements with low amplitude (10–20°) and high acceleration (3,000–4,000°/s) on the patient's head to the left and right in a horizontal movement. The latency with which eye movements occurred, after the head was accelerated, was detected. The correlation between head and eye movements was registered as gain (= quotient eye speed ÷ head speed), with values below 0.8 being considered conspicuous. The gain asymmetry (GA) was calculated using the following formula:

$$GA=\frac{\left(gain\;left)-(gain\;right\right)}{gain\;left\;+\;gain\;right}\;x100\%$$

As suggested by Patscheke et al. (38), gain asymmetry of ≥8% was rated as conspicuous.

### Caloric Irrigation Test

Before irrigation, ear inspection was performed to ensure the integrity of the tympanic membrane, and a recording of preexisting horizontal spontaneous nystagmus was obtained. The patient was lying down, and the upper body was raised by 30° (resulting in a vertical position of hSCC). The patient wore video glasses with an integrated camera that recorded the eye movements using the “Visual Eyes 525” video oculography system, which were passed on to the “OtoAccessTM” software. According to Hallpike (39), the caloric response to bithermal stimulation was recorded when flushing with 100 ml of 44 or 30°C warm water for 30 s each. Pauses of at least 5 m were carried out between the rinses (33). Nystagmus reaction was recorded for 80 s, and SPV was determined from a 20-s interval. To measure the caloric response recorded in the implanted ear, the sum of SPV of the cold and warm irrigation was calculated.

For quantitative evaluation, values below 5°/s were counted as loss of the vestibular organ. With a total SPV in the implanted ear below 10°/s, hypoexcitability of this side was determined. Values above 40°/s were considered to be hyperexcitable. The degree of side difference (SD) was evaluated using the JONGKEES formula (40):

$$SD=\frac{\left(mSPV\;right\;30^{{}^{\circ}}C\;+\;mSPV\;right\;44^{{}^{\circ}}C\right)\;-\;(mSPV\;left\;30^{{}^{\circ}}C\;+\;mSPV\;left\;44^{{}^{\circ}}C)}{mSPV\;right\;30^{{}^{\circ}}C\;+\;mSPV\;right\;44^{{}^{\circ}}C\;+\;mSPV\;left\;30^{{}^{\circ}}C\;+\;mSPV\;left\;44^{{}^{\circ}}C}\;x100\%$$

As suggested by Reiß et al. (41), SD of more than 20% was considered conspicuous.

### Data Analysis

The pre- and post-operative results of the test battery were compared and examined for changes.

The postoperative results of caloric irrigation test and vHIT test in the implanted patients were analyzed with regards to the insertion depth of the electrode and the electrode design. Data were tested for normal distribution using the Shapiro–Wilk test. To measure differences in outcomes from pre- to postoperative, paired-samples *t*-test was used when the distribution was normal, and Wilcoxon test was used when the distribution was not normal. To investigate the influence on the development of postoperative vertigo, Fisher's exact test or descriptive statistics was used when the number of cases was less than five patients. To test the influence of nominal test battery scores, we used logistic regression (IBM® SPSS® Statistics, version 27).

## Results

### Questionnaire

Patients were grouped according to the questionnaire outcome reflecting their vertigo symptoms. As shown in Table 2, 13 patients were assigned to group 0 (before and after surgery, no vertigo), 11 to group 1 (before surgery, no vertigo; after surgery, vertigo), three to group 2 (before surgery, vertigo; after surgery, no vertigo), and two to group 3 (before and after surgery, vertigo). Thus, vertigo associated with cochlear implant treatment occurred newly in 11 of 24 cases (45.8%). The patients were classified into groups based on their symptoms, and only in group A was the vertigo most likely to have a vestibular origin. In group B, only one possible cause for a vestibular disorder was identified; in group C, there was none. Five patients had preoperative vertigo (groups 2 and 3). Of these, three were assigned to group A, one to group B, and one to group C. One of these patients (n17) consistently reported vertigo symptoms in all questionnaires, with no change in quality. Patient n3 did not report a recurrence of vertigo until the 6-month questionnaire, with the quality of vertigo remaining the same. Nine patients had a new onset of vertigo within the first week after implantation, and in two patients, the vertigo occurred newly 6 months after the initial fitting (group 1). Of these, five patients were counted as group A, three patients as group B, and three patients as group C. Thus, in eight of 11 cases (72.7%), a vestibular or at least possibly vestibular cause for the postoperative new-onset vertigo could be identified on the basis of the questionnaire data. Respectively, eight out of 24 patients (33%) had new vertigo with a vestibular or possibly vestibular cause.

**Table 2:** Chronological overview of the occurrence of vertigo (cases without any occurrence of vertigo excluded).

**Patient ID**	**Vertigo group**	**Etiology**	**Suspected origin** ** of vertigo**	**Pre-op**	**1W**	**First fit**	**3M**	**6M**
n8	1	Unknown	B		X	X	X	
n12	1	Unknown	A		X			
n14	1	Congenital	A					X
n15	1	Congenital	A		X			
n18	1	Unknown	B		X	X	X	X
n23	1	Congenital	C		X	X	X	
n27	1	Unknown	C		X			
n28	1	Unknown	B		X	X	X	X
n30	1	Unknown	A		X			
n31	1	Sudden deafness	A		X			
n32	1	Ménière's disease	C					X
n13	2	Unknown	C	X				
n16	2	Ménière's disease	B	X				
n24	2	Congenital	A	X				
n3	3	Apoplexy	C	X				X
n17	3	Unknown	A	X	X	X	X	X

*Vertigo group 1 = –/+, 2 = +/+, 3 = +/–, vertigo (+) pre-operative/post-operative, suspected origin of vertigo*.

*A, profound suspicion of vestibular origin; B, potential vestibular origin; C, suspicion of central origin; Pre-op, 1 day before surgery; 1W, 1 week after surgery; First fit, at the time of initial adjustment; 3M, 3 months after initial adjustment; 6M, 6 months after initial adjustment; X, patient-reported vertigo*.

### Vestibular Function Tests

The results obtained from the Romberg test and optokinetic test showed normal results preoperatively and postoperatively in all 29 patients.

### Unterberger Test

Already preoperatively, six patients, all of whom had no complaints of vertigo according to the questionnaire evaluation (21%), showed a conspicuous test result. Four of the six patients (67%) deviated to the side to be implanted. Postoperatively, the number of pathologic test results increased to 10 (34%), with eight of these 10 patients (80%) deviating to the implanted side. Thus, this number of patients showing deviation to the implanted side increased from four to eight. This difference was not statistically significant in the Wilcoxon test (*Z* = −1.826; *p* = 0.068). An overview of the conspicuous results of the Unterberger test is shown in Table 3. All patients who did not have normal findings preoperatively also had conspicuous findings postoperatively. The four patients who had new-onset conspicuous results in the Unterberger test all showed a deviation to the implanted side. Three of these patients also reported new-onset vertigo in the questionnaire. There was no statistically significant correlation between the results and the incidence of postoperative vertigo in binary logistic regression as shown in Table 4.

**Table 3:** Unterberger test, cases with conspicuous results only, pre- and post-operative (for information on vertigo group and origin of vertigo, see Table 2).

**Vertigo group**	**Suspected origin** ** of vertigo**	**Patient ID**	**Preoperative rotation** ** (degrees)**	**Postoperative rotation** ** (degrees)**
0		n10		45 (–)
0		n19		75 (+)
0		n21	80 (+)	80 (+)
0		n25	70 (+)	70 (+)
0		n26	90 (+)	90 (+)
1	A	n15		90 (+)
1	B	n18	50 (+)	50 (+)
1	B	n28	90 (–)	90 (–)
1	C	n23		80 (+)
1	C	n32	90 (–)	60 (+)

*+, rotation toward the implanted side; –, rotation away from the implanted side*.

**Table 4:** Correlation of postoperative test battery scores and development of postoperative vertigo (logistic regression).

	***B***	**SE**	**Wald**	***p***	**Odds ratio**	**95% CI for odds ratio**
						**Lower bound**	**Upper bound**
Unterberger deviation toward Implanted side postoperative	−0.009	0.033	0.066	0.797	0.991	0.929	1.058
vHIT gain postoperative Implanted side	27.24	19.789	1.895	0.169	6.76E + 11	0	4.73E + 28
vHIT GA postoperative	0.289	0.206	1.965	0.161	1.335	0.891	2
Caloric SD postoperative Implanted side	−0.027	0.047	0.323	0.57	0.974	0.888	1.068
Caloric SPV postoperative Implanted side	−0.096	0.111	0.749	0.387	0.909	0.731	1.129

*Degrees of freedom were 1 for all Wald statistics*.

### Subjective Visual Vertical

When measuring SVV, only one study participant (3.4%), who did not perceive vertigo at any time, had an atypical result both pre- and post-operatively. Otherwise, all other results were regular at both test visits. Thus, except for this one case, there was no evidence for utricle or central damage to balance.

### Video Head Impulse Test

The vHIT could not be performed postoperatively in one case because the patient had massive neck pain with accompanying vertigo symptoms. Preoperative testing was also missing in one patient who did not have vertigo at any time and had an unremarkable finding on postoperative vHIT. The findings of the implanted side as well as the comparison with the opposite side were evaluated and are shown in Table 5.

**Table 5:** vHIT test, conspicuous results only (for information on vertigo group and origin of vertigo, see Table 2).

**Vertigo group**	**Suspected origin** ** of vertigo**	**Patient ID**	**Gain,** ** preoperative**	**Gain,** ** postoperative**	**GA, preoperative** ** [%]**	**GA, postoperative** ** [%]**
0		n1			17 (+)	
0		n4				9 (+)
0		n5			14 (–)	30 (–)
0		n7		0.8	56 (–)	14 (–)
0		n10			12 (+)	22 (+)
0		n11				16 (+)
0		n19			12 (+)	
0		n20			13 (+)	11 (+)
0		n26				15 (+)
1	A	n12			14 (+)	15 (+)
1	A	n14				8 (+)
1	A	n15			10 (–)	18 (–)
1	A	n30	0.6		13 (+)	8 (+)
1	B	n8			12 (+)	
1	B	n28			15 (+)	13 (+)
1	C	n23			17 (–)	
1	C	n27				8 (–)
1	C	n32			12 (–)	
2	A	n24	0.7	0.8		14 (–)
2	C	n13				9 (–)
3	A	n17			20 (+)	12 (–)
3	C	n3				16 (–)

*GA, gain asymmetry; +, implanted side; –, opposite implanted side*.

### Gain Implanted Side

Two pre- and post-operative patients had a conspicuous gain (<0.8). One patient, who had vertigo with a vestibular cause preoperatively and who no longer reported vertigo postoperatively, had conspicuous values at both times. A second patient had pathologic gain only postoperatively, although this participant reported no vertigo in the questionnaire. Another patient, who had vertigo postoperatively with a most likely vestibular origin, showed a conspicuous gain preoperatively only, while the postoperative value was normal. The preoperative mean gain of the implanted side was 1.0 (median: 1.0; SD ± 0.2) and did not change postoperatively (median: 0.9; SD ± 0.17), with an interval from 0.8 to 1.5. There was no significant difference in gain values before and after implantation [*t*_(26)_ = 0.383; *p* = 0.705].

### Gain Asymmetry

Preoperatively, 14 cases (50%) with irregular GA (≥8%) with 9/14 (64%) lower gain on the implanted side were observed. Postoperatively, in 17 patients (61%), irregular GA, with 9/17 (53%) lower gain on the implanted side, occurred. Irregular values were measured at both times in patients with and without vertigo. Four patients noticed postoperative vertigo *and* had a conspicuous GA with lower gain on the implanted side. Within this group of patients, a vestibular origin was very likely in three and possible in one patient. However, there were also five patients with conspicuous GA who did not complain about vertigo.

The GA values worsened in seven cases (25%) without being statistically significant in Wilcoxon's test (*Z*= *-*0,84; *p* = 0.933). We found no correlation between gain or GA with the self-reported occurrence of postoperative vertigo in binary logistic regression as shown in Table 4. Preoperatively, the interval of GA ranged from 0 to 56%, with a mean of 10.5% (median: 8.5%; SD ± 10.48%). Postoperatively, the interval of GA ranged from 1 to 30%, with a mean of 9.9% (median: 8.5%, SD ± 6.92%).

### Caloric Irrigation Test

The test was performed preoperatively in all patients and postoperatively in 26 patients. An overview of cases with conspicuous results for both test intervals is given in Table 6.

**Table 6:** Caloric irrigation test result, slow phase velocity, and side difference (SD) conspicuous results only (for information on vertigo group and origin of vertigo, see Table 2).

**Vertigo group**	**Suspected origin** ** of vertigo**	**Patient ID**	**Interpretation** ** preoperative**	**Interpretation** ** postoperative**	**SD preoperative** ** (%)**	**SD postoperative** ** (%)**
0		n1	PH	PH	25 (+)	47 (+)
0		n4	PH	N/A		N/A
0		n7	PH	PH		46 (–)
0		n20	PH	N/A		N/A
0		n21	PH	PH		54 (–)
0		n25	PH	N/A	26 (+)	N/A
0		n29	PH	PH		42 (+)
1	A	n12	PH	PH	30 (–)	
1	A	n14	UN	FA		95 (+)
1	A	n30	UN	PH		
1	A	n15	PH	PH	23 (–)	45 (–)
1	B	n18	PH	UN		47 (+)
1	C	n32	PH	PH		37 (+)
2	A	n24	UN	UN	56 (+)	
2	B	n16	UN	PH	58 (+)	28 (+)
2	C	n13	PH	PH	28 (+)	
3	A	n17	PH	PH		
3	C	n3	HE	PH		

*PH, physiological; HE, hyperexcitability; UN, hypoexcitability; FA, failure; N/A, no answer; +, implanted side worse response; –, opposite implanted side worse response*.

**Figure 2:**
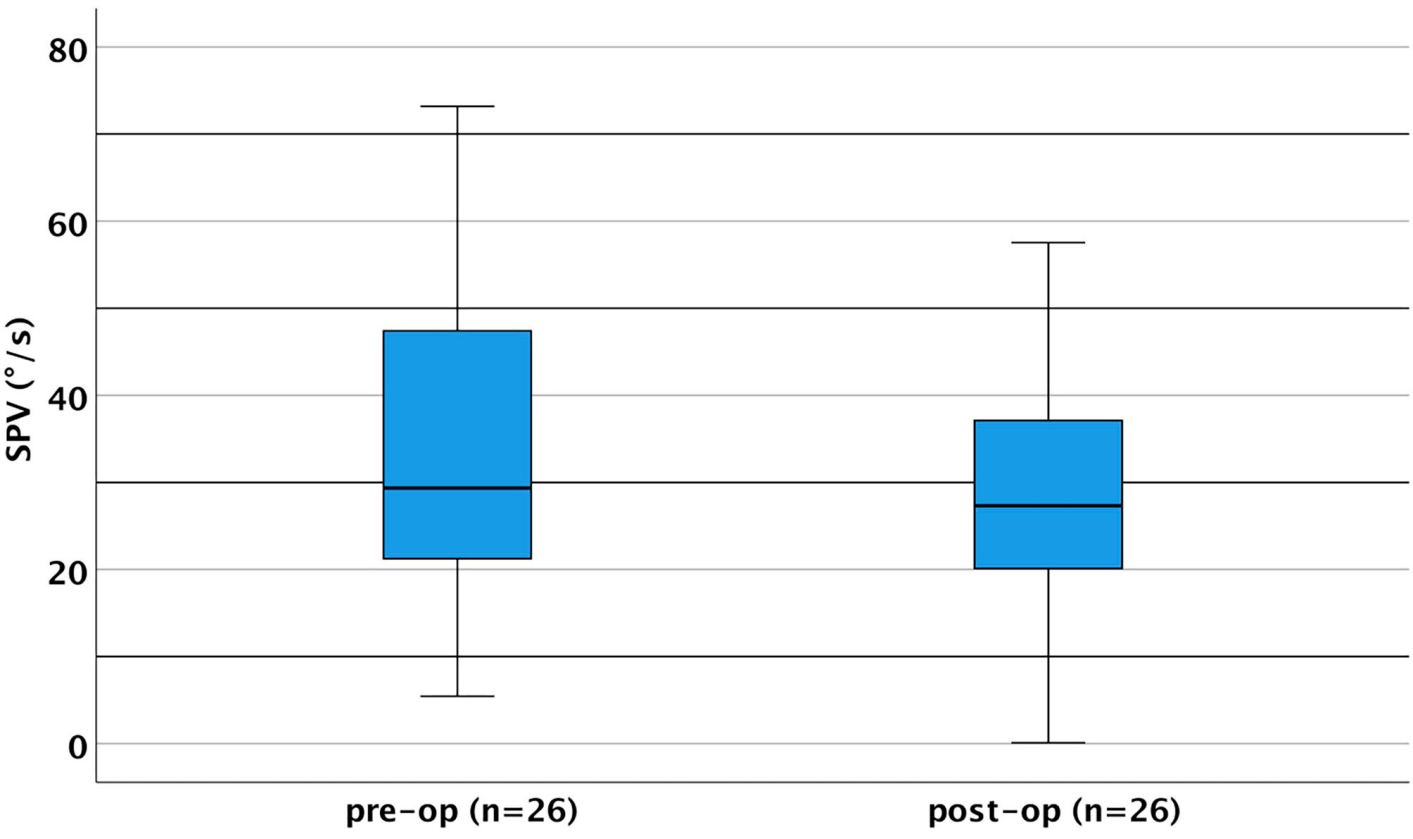
Caloric slow-phase velocity (SPV; sum of cold and warm irrigation) pre- and post-operatively of the implanted side (boxplots). SPV ranged preoperatively from 5.44°/s up to 73.19°/s (mean = 32.78 ± 18.50°/s) and postoperatively from 0.1°/s up to 57.53°/s (mean = 28.74 ± 15.41°/s). SPV was approximately normally distributed for both intervals as assessed by the Shapiro–Wilk test (preoperatively, *p* = 0.242; postoperatively, *p* = 0.565).

Overall, the SPV of the implanted side worsened in 20/26 (77%) patients. The average caloric response in the implanted side was reduced from 32.78°/s (median: 29.82°/s; SD ± 18.5) preoperatively to 28.74°/s (median: 27.33°/s; SD ± 15.4) postoperatively (see Figure 2 for further details). There was no statistically significant difference in SPV before and after implantation [*t*_(25)_ = 1.290, *p* = 0.209].

Preoperatively, hypoexcitability of the lateral semicircular canal of the implanted side was found in four patients and hyperexcitability in one. Postoperatively, a complete loss of function was obvious in one case. After implantation, in two patients who showed hypoexcitability and hyperexcitability preoperatively, this was no longer detectable. Based on the questionnaire results, all of these patients showed postoperative signs of vertigo (groups 1 and 3). One group 3 patient who showed a hyperexcitable lateral semicircular canal preoperatively dropped to normal SPV values postoperatively despite ongoing vertigo problems. In one (n14) of the four patients who were considered to be hypoexcitable preoperatively, a SD of 95% was found postoperatively. This individual belonged to group 1, and an evaluation of the questionnaire suggested a vestibular cause. One patient (n18) showed physiological values preoperatively and complained about the onset of vertigo postoperatively, while hypoexcitability was evident, which was also confirmed by SD. A potential vestibular origin was revealed by the questionnaire. In group 1, however, there was one patient (n30) who showed hypoexcitability of the SD side preoperatively, which was not confirmed in the postoperative testing, although the vertigo appeared to be of vestibular origin.

Three of five patients with preoperative vertigo (groups 2 and 3) already showed abnormal results preoperatively, which could indicate a preexisting lesion of the hSCC. The questionnaire confirmed a potential vestibular origin in two of these three cases. One patient (n24) showed hypoexcitability, confirmed by a conspicuous SD that persisted postoperatively, with the SD disappearing. In another case (n16), the preoperative hypoexcitability of the implanted side was also confirmed by a conspicuous SD, which persisted postoperatively. However, the hypoexcitability of the implanted side could no longer be measured postoperatively (as can be seen in Table 7).

**Table 7:** Result categories of caloric response test (pre- and post-operative) related to vertigo group (for information on vertigo group, see Table 2).

	**Preoperative vertigo group**					**Postoperative vertigo group**				
	**0**	**2**	**1**	**3**		**0**	**2**	**1**	**3**	
**Interpretation**	**No postoperative vertigo**		**Postoperative vertigo**		**Total**	**No postoperative vertigo**		**Postoperative vertigo**		**Total**
Physiological	13 (100%)	1 (33%)	9 (82%)	1 (50%)	24	10 (77%)	2 (67%)	9 (82%)	2 (100%)	23 (79%)
Hyperexcitable	0	0	0	1 (50%)	1	0	0	0	0	0
Underexcitable	0	2 (67%)	2 (18%)	0	4	0	1 (33%)	1 (9%)	0	2 (7%)
Failure	0	0	0	0	0	0	0	1 (9%)	0	1 (4%)
Not performed	0	0	0	0	0	3 (23%)	0	0	0	3 (10%)
Total	13 (100%)	3 (100%)	11 (100%)	2 (100%)	29 (100%)	13 (100%)	3 (100%)	11 (100%)	2 (100%)	29 (100%)

*Postoperative vertigo, occurrence of self-reported vertigo at any interval*.

### Side Difference

Conspicuous values of the SD occurred both pre- and post-operatively in all groups. We noticed that the values of our quantitative analysis, based on conspicuous values of excitability, were confirmed by the SD but that conspicuous values of SD also occurred in patients without vertigo. SD related to the implanted side (characterized by decreased caloric response) occurred preoperatively in five patients (17%) and to the non-implanted side in two patients (7%). Postoperatively, SD related to the implanted side was found in six patients (23%) and related to the non-implanted side in three patients (12%). Both the change in SD in the implanted side (*p* = 0.281) and in the non-implanted side (*p* = 0.071) was not statistically significant. There was no difference in SD before and after implantation [*t*_(8)_ = −1.155; *p* = 0.281]. We found no correlation between the SPV values or the SD and the occurrence of self-reported vertigo in binary logistic regression as shown in Table 4. Preoperatively, the SD was between 0 and 58%. The mean value was 15% (median 11%, standard deviation ±14%). Postoperatively, the interval of the SD was between 0 and 95%, with a mean of 21% (median 9 ± 24%).

### Insertion Angle

**Figure 3:**
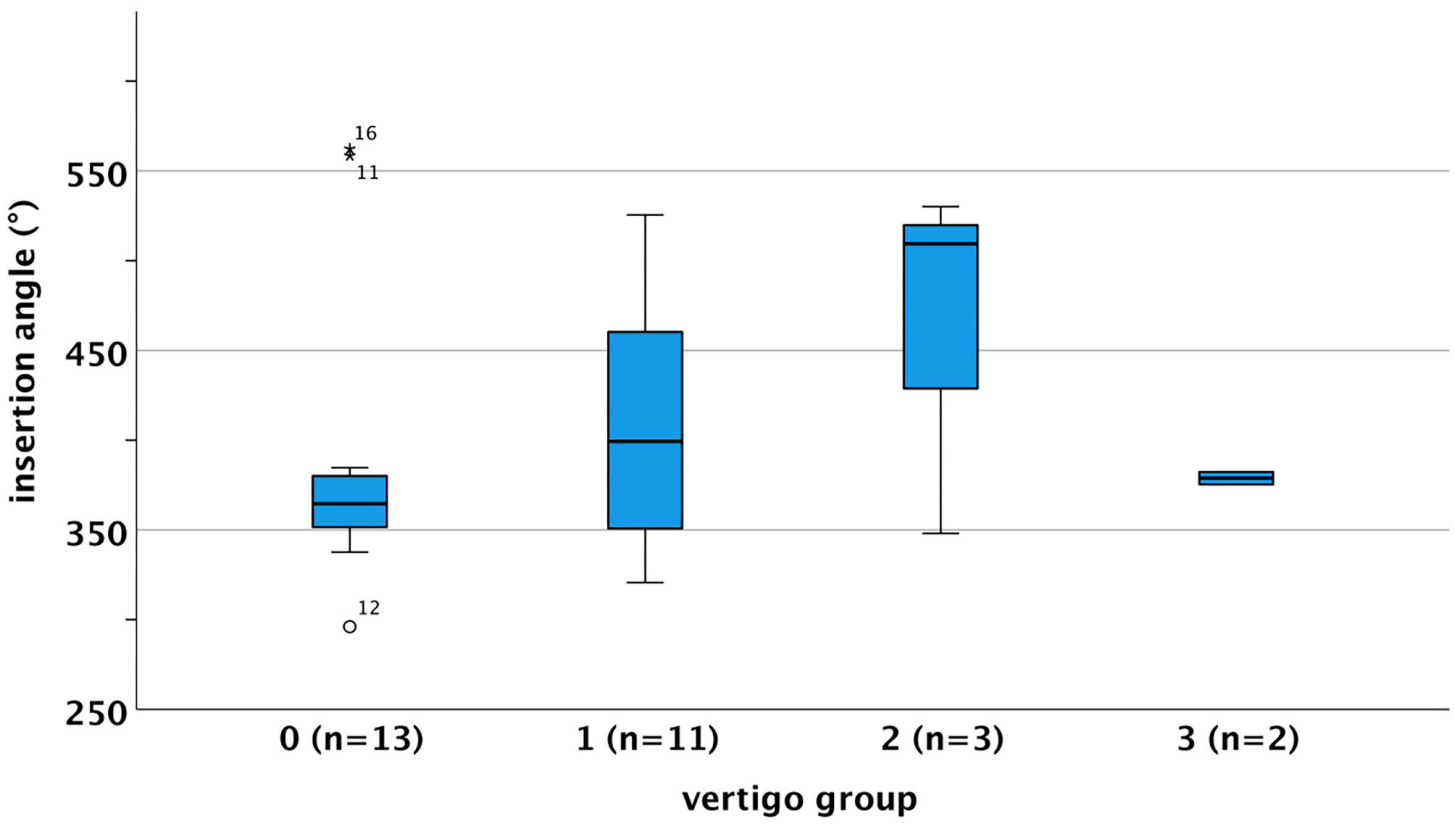
Insertion angle (IA) distributions depending on vertigo group [boxplot, 0 = –/–, 1 = –/+, 2 = +/+, 3 = +/–, vertigo (+) pre-op/postoperative]. The IA ranged in vertigo group 0 from 296.10° up to 562.24° (mean = 389 ± 82.90°), in vertigo group 1 from 320.71° up to 525.5° (mean = 409.21 ± 72.29°), in vertigo group 2 from 348.11° up to 530.18° (mean= 462.58 ± 99.67°), and in vertigo group 3 from 375.39° up to 382.31° (mean = 378.85 ± 4.89°). IA was approximately normally distributed for vertigo group 1 (*p* = 0.192) and vertigo group 2 (*p* = 0.199), but not for vertigo group 0 (*p* = 0.001) as assessed by the Shapiro–Wilk test. As vertigo group 3 consisted of only two cases, no normal distribution could be tested.

Evaluation of the postoperative CT images showed an average insertion angle of 404° for the total collective. The individual values were 389° in the vertigo group 0, 409° in group 1, 463° in group 2, and 379° in vertigo group 3 (see Figure 3). Patients with an insertion angle of <430° (IA category u) reported vertigo postoperatively in nine of 20 cases (45%), whereas in the group with insertion of 430° or more (IA category o), four of eight patients reported vertigo (see Table 8).

**Table 8:** Relation of insertion angle category and vertigo group (for information on vertigo group, see Table 2).

		**Vertigo group**				
		**0**	**2**	**1**	**3**	**Proportion of patients with postoperative vertigo within the IA group**
**IA category**	**Number of patients**	**Postoperative no vertigo**		**Postoperative vertigo**		
U	20	10	1	7	2	9/20 (45%)
O	8	2	2	4	0	4/8 (50%)
Not measurable	1	1	0	0	0	0/1 (0%)
Total	29	13	3	11	2	

*IA, insertion angle; U, <430°; O, ≥430°*.

A chi-square test was used to compare the occurrence of postoperative vertigo and insertion angle. As there were two expected cell frequencies below 5, Fisher's exact test was applied instead. The results show no significant relation between the occurrence of postoperative vertigo and insertion angle.

### Electrode Design

**Figure 4:**
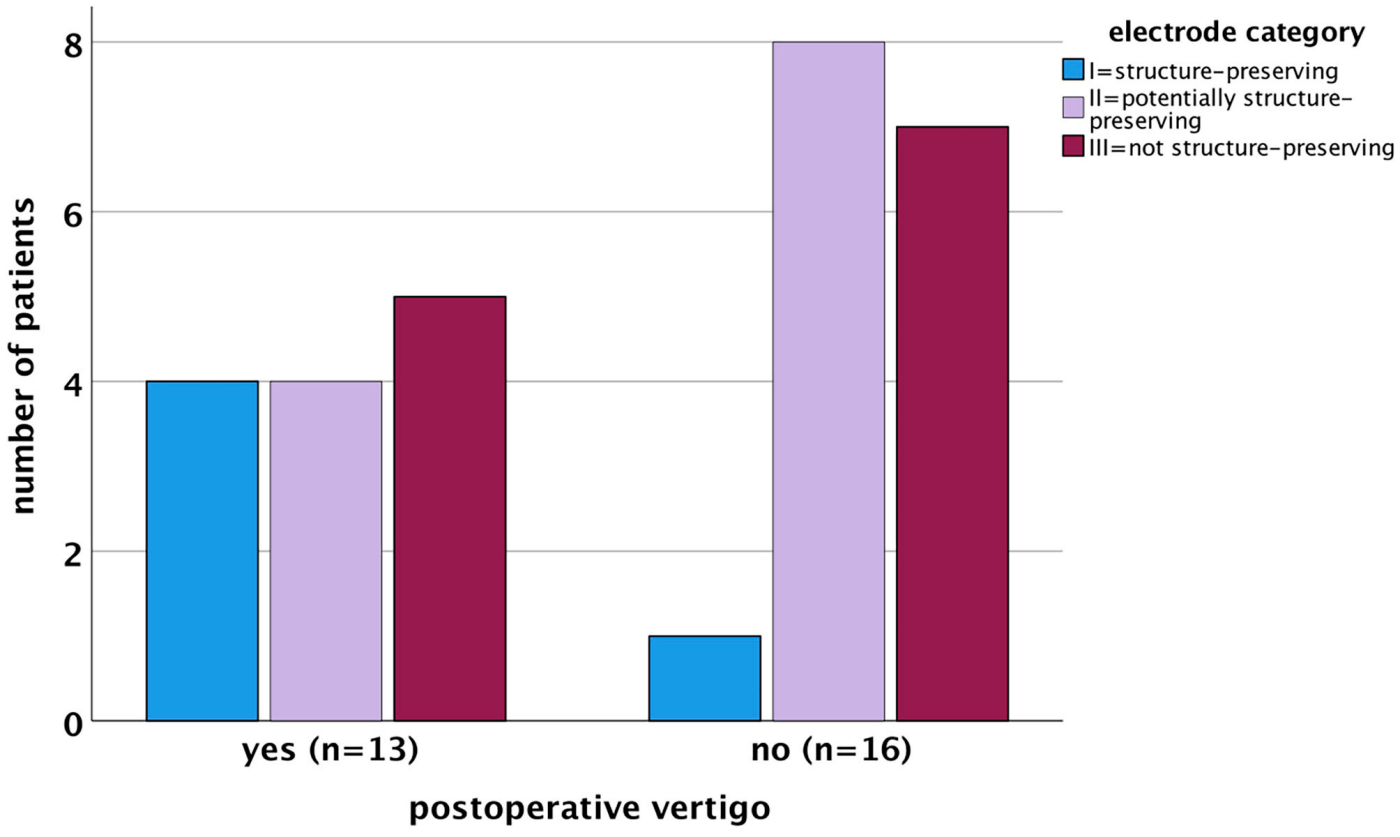
Correlation between the occurrence of postoperative vertigo and electrode categories (bar chart). The electrode categories were not normally distributed in patients with postoperative vertigo (*p* = 0.007) as well as in patients without postoperative vertigo (*p* = 0.001) as assessed by the Shapiro–Wilk test.

The electrode carriers were categorized into three groups according to their design–properties, as depicted in Table 1. A total of five out of 29 patients (17%) were assigned to electrode category I (structure-preserving), as shown in Table 9. After implantation, two out of five (40%) patients newly developed vertigo (group 1). According to the questionnaire, one patient had vertigo with a possible vestibular cause (group B); the other suffered from vertigo due to most likely non-otogenic reasons (group C). Two patients in group 3, who were supplied with electrodes of electrode category I, had vertigo after implantation. However, they already complained about vertigo before operation. Electrode category II (potentially structure-preserving) was assigned to 12 out of 29 (41.4%) patients. Here postoperative vertigo occurred in 5/12 (41.6%) patients, with new onset in all cases (vertigo group 1). All patients belonged to group A or B with assured vestibular or possibly vestibular cause of vertigo. There were 12 (41.4%) patients in electrode category III (not structure-preserving). In four out of these 12 CI users (33.3%), vertigo occurred newly after implantation (vertigo group 1). Based on the questionnaire, we suspected a vestibular cause (group A) in two patients and a non-vestibular cause (group C) in the other two. Due to the small number of cases within the electrode groups, statistical tests were not applied for the comparison (for the results of the descriptive statistics, see Figure 4).

**Table 9:** Occurrence of vertigo within electrode categories (for information on vertigo group, see Table 2).

		**Vertigo group**				
		**0**	**2**	**1**	**3**	**Proportion of patients with postoperative vertigo within the electrode group**
**Electrode category**	**Number of patients**	**Postoperative no vertigo**		**Postoperative vertigo**		
I	5	1	0	2	2	4/5 (80%)
II	12	5	2	5	0	5/12 (42%)
III	12	7	1	4	0	4/12 (33%)
Total		13	3	11	2	

*I, structure-preserving; II, potentially structure-preserving; III, not structure-preserving*.

### Low-Frequency Residual Hearing

**Figure 5:**
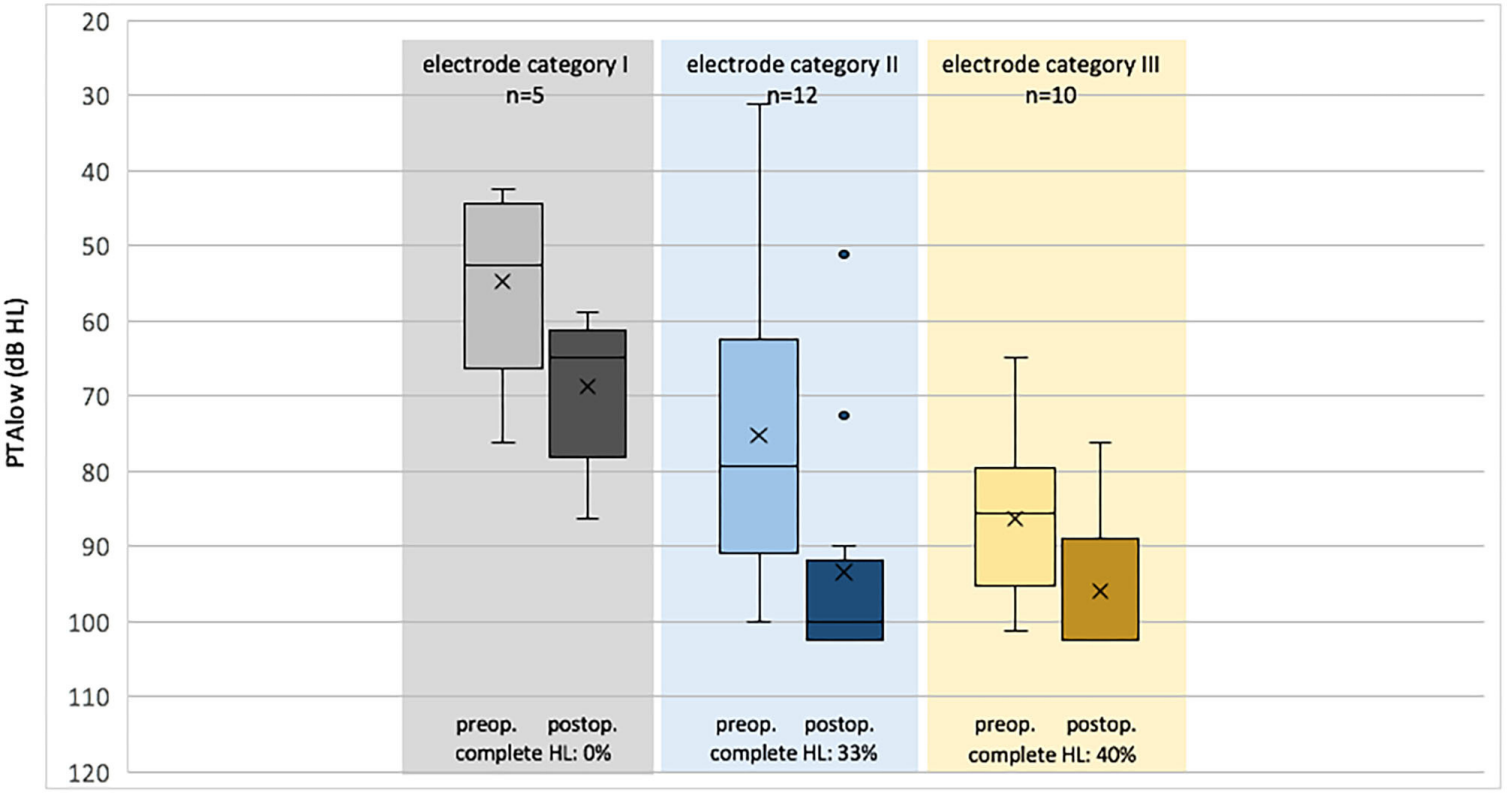
Pre- and post-operative PTAlow (averaged unaided air conduction threshold, 125, 250, 500, and 1,000 Hz, boxplots) grouped by electrode category (I, structure-preserving; II, potentially structure-preserving; III, not structure-preserving).

After CI provision, eight study participants lost their residual hearing and were excluded. Among these, four cases belonged to electrode category II (2× CI 532, 2× FLEX28), and four cases belonged to electrode category III (1× HiFocus MidScala, 3× CI512), corresponding to a percentage of residual hearing loss of 15% per group. In addition, two cases were excluded from evaluation because of preoperative deafness. Therefore, 19 cases were available for analysis of hearing preservation (Figure 5).

In electrode category I, PTAlow ranged preoperatively from 43 dB HL up to 76 dB HL (mean 55 ± 13.05 dB HL) and postoperatively from 59 dB HL up to 86 dB (mean 69 ± 10.47 dB HL), with a mean difference of 14 dB; in electrode category II, it ranged preoperatively from 31 dB HL up to 95 dB HL (mean 70 ± 21.04 dB HL) and postoperatively from 51 dB HL up to 103 dB HL (mean 89 ± 18.34 dB HL), with a mean difference of 19 dB; in electrode category III, it ranged preoperatively from 65 dB HL up to 101 dB HL (mean 86 ± 11.31 dB HL) and postoperatively from 76 dB HL up to 103 dB (mean 96 ± 9.35 dB HL), with a mean difference of 10 dB. The PTAlow difference was normally distributed, as assessed by the Shapiro–Wilk test (*p* = 0.125).

Over all categories, the PTAlow before and after implantation was statistically different [paired-samples *t*-test, *t*_(18)_ = −5.288, *p* < 0.001]. Split to the electrode category, the same was observed for electrode category I [*t*_(4)_ = −5.199, *p* = 0.007] and category II [*t*_(7)_ = −4.134, *p* = 0.004], but not for category III [*t*_(5)_ = −1.852, *p* = 0.123].

After calculating the PTAlow difference, testing for normal distribution, testing for variance homogeneity, and removing outliers, we conducted a one-way ANOVA to investigate whether there was a difference in PTAlow difference depending on the electrode categories. The PTAlow difference was statistically significant for the different electrode groups, *F*_(2,23)_ = 284, *p* < 0.001. The Tukey *post-hoc* test showed a significant difference (*p* < 0.001) in PTAlow between electrode groups 2 and 3 [10.4, 95% CI (1.17, 19.61)], while the difference between electrode groups 1 and 2 [*p* = 0.435; 5.7, 95% CI (−5.7, 17.07)] and electrode groups 1 and 3 [*p* = 0.573; 4.7, 05% CI (−6.86, 16.26)] was not significant.

## Discussion

The occurrence of vertigo as a postoperative complication after cochlear implantation has been described in several studies (1, 7, 42). The risk of this has been reported to range in incidence from 12 to 74% (43, 44). In the study presented here, the incidence of new vertigo after CI surgery was 45.8% (11/24 patients). Despite numerous efforts to identify triggers for vertigo and to introduce improvements in CI surgery protocols, vertigo is still considered a common side effect (11).

### Romberg Test

Since no patient in our study had a conspicuous result in the Romberg test, it can be concluded that neither preoperatively nor postoperatively was a central lesion present. Kaczmarczyk et al. (45) who used the Romberg test to assess gait stability before and after cochlear implantation, also found no increased stance or gait instability postoperatively.

### Unterberger Test

Abnormal rotations were detected in patients of the present cohort with and without vertigo symptoms using the Unterberger test. However, the weak sensitivity and specificity of this test was already described in 1944 by Winkler (46). The authors concluded that a negative result of the test could not exclude vestibular dysfunction, and a positive result could not confirm it. Similarly, a more recent study by Hickey et al. (47) reported no significant Unterberger test result difference between patients with and without vestibular pathology. Our results did show that patients with deviation in the Unterberger test mostly turned to the implanted side postoperatively, and 75% (3/4 patients) of cases complained of vertigo. Nevertheless, as reported in the mentioned previous studies, there were also patients with conspicuous results who were asymptomatic. Thus, we conclude that the test is not sufficiently informative with respect to vestibular damage.

### Subjective Visual Vertical

Gnanasegaram et al. (48) demonstrated conspicuous values in the SVV test ~1 year postoperatively in 45% of patients after cochlear implantation. In the study presented here, these results could not be confirmed, neither in patients with nor without vertigo. The reason for this discrepancy could be the fact that, within the 6-month follow-up period, compensation of otolith function had been achieved, which according to Böhmer (34) can occur within weeks to a few months after damage. On the other hand, this would again contradict the results of Gnanasegaram et al. (48) who showed pathologic results despite a longer observation period, although this could also be caused by increased vestibular damage within the studied patient group.

In their study of 12 patients, le Nobel et al. (49) demonstrated that conspicuous SVV results resulted at all time points before CI surgery, 1 week and 1 month postoperatively, but did not change significantly. According to Sun et al. (50), the SSV test correlates with the asymmetry ratio of the oVEMP but is easier to perform and also less expensive, which is why it was recommended by the author for measuring the utricle function of patients at the time of vertigo.

### Optokinetic Testing

According to Yetiser et al., optokinetic testing can detect evidence of central disorders, particularly cerebellar damage and brainstem damage. Because none of the patients in this study had postoperative abnormalities, it can be concluded that cochlear implantation did not result in central lesions (51). These could be excluded preoperatively as a cause of vertigo among all patients in vertigo classes 2 and 3. No conspicuous values were measured for patient n3 either, who indicated an apoplexy as the etiology of the hearing loss.

### Video Head Impulse Test

When considering the vHIT results of this study, it is noticeable that no conspicuous decrease in gain (gain below 0.8) in the implanted side was detected postoperatively in any patient with postoperative vertigo. In contrast, two patients without postoperative vertigo (n7 and n24) had pathologic decrease in gain in the implanted side. Furthermore, it was noticeable that, in patients with preoperative vertigo, both the preoperative mean gain in the implanted side, 1.0 ± 0.2, and in the non-implanted side, 1.1 ± 0.3, were within the normal range. Considering the patients who suffered from vertigo postoperatively, the mean gain of 0.98 ± 0.2 in the implanted side and 0.95 ± 0.15 in the non-implanted side was similar, although a conspicuous result of the implanted side would be expected. In the *t*-test for paired samples, there was no difference in gain values after implantation between both sides [*t*_(27)_ = 0.376, *p* = 0.710].

Similar to previous studies, cases with conspicuous values were identified in the caloric test, while the vHIT gain parameter was in the physiological range, although both tests measure the function of the hSCC (38). Blödow et al. (52) assumed that, in the case of peripheral vestibulopathy, as suspected after implantation, the caloric irrigation test would show conspicuous values more frequently than vHIT. Dagkiran et al. (53) examined 42 CI patients 3 days and 3 months postoperatively and reported that the number of patients with deteriorated vHIT results decreased from 16.6 to 2.3%. Similar observations were described by Jutila et al. who examined patients with acute vestibular loss using vHIT on day 3 and at 3 months after the occurrence of vestibular symptoms. There was a highly significant improvement in gain from deteriorated to normal values as well as a decrease in previously existing asymmetry (54). Ibrahim et al. (55) demonstrated that cochlear implantation had no significant effect on the outcome of vHIT, consistent with our observations.

Furthermore, it became obvious from the results of the present study that the GA parameter was most frequently conspicuous. We suspected that the reason for this could be that the thresholds for deviation used in this study (>8/ ≤ 8%) were set too low and could also occur physiologically. We examined whether patients with vertigo had a higher GA value but could not find any difference between patients with and without self-reported vertigo. In general, thresholds of deterioration are controversially discussed in the literature and range from 2 to 20% (14, 56–58). In summary, GA was not related to subjectively reported vestibular status. Therefore, evaluation of gain asymmetry in terms of conspicuous or physiological category was not decisive.

In addition, an evaluation of vHIT catch-up saccades was carried out, which are considered typical of peripheral lesions (59). However, we found no association of catch-up saccades with the occurrence of vertigo. Patscheke et al. (38) analyzed the occurrence of vHIT catch-up saccades in 171 patients suffering from vertigo and showed that the sensitivity of the vHIT for detecting a peripheral–vestibular disorder was low and that the two (peripheral) parameters “gain” and “catch-up saccades”—contrary to expectations—were only conspicuous to a small extent (22%) in the same patients.

### Caloric Irrigation Test

The caloric irrigation test is a long-established measurement technique that has been used in multiple studies to investigate the function of hSCC after cochlear implantation (9, 42, 60, 61). It has been described frequently that cochlear implantation has a significant impact on the outcome of this functional test (15, 55). However, there are also controversial reports, e.g., Colin et al. (9) reported no significant correlation between caloric irrigation test results and individual vertigo symptoms. This observation was confirmed by Zeng et al., where 18 patients were tested preoperatively and at 1 week and 1 month postoperatively (61).

In the present study, SPV decreased slightly, about 4°, within the total group (pre-operative/post-operative comparison) without statistical significance. Pre-operatively and post-operatively, hypoexcitability, vestibular loss, and/or SD larger than 20% with lower caloric response in the implanted side were found in 31% of the whole collective. This observation is in agreement with the results of Ibrahim et al. (55), who reported 39.5% pathologic cases preoperatively and 28% postoperatively.

Concerning the criteria for classification of pathologic results, SPV values below 10°/s were categorized as hypoexcitability and those SPV below 5°/s as vestibular loss [following the suggestions of Holinski et al. (2)]. Alternatively, SPV values below 3°/s or even 7°/s were discussed as markers for hypoexcitability, with SPV = 0°/s as indicator of vestibular failure (62, 63). Related to the present study, hypoexcitability was present in all patients with self-reported pre- or post-operative vertigo (Table 6). Since SPV was above 3°/s in these cases, we consider this limit to be too low. A single individual in this group even showed SPV above 7°/s. We therefore consider a limit of SPV <10°/s to be appropriate to characterize the irregular function of hSCC.

Different limits for physiologic SD (or canal paresis) have likewise been discussed. The generally accepted limit of physiological SD is between 20 and 25% (41, 61, 64, 65).

Remarkably, SPV and SD outcomes had improved in two cases after surgery (n16, n30). On the one hand, this could be due to the limited reliability of the test. On the other hand, an improvement of vestibular function after CI is reported in the literature, which the authors attributed to the chronic stimulation of the labyrinth (9).

### Correlation of Vertigo Test Results With Self-Reported Vertigo

In conclusion, the efforts to objectify self-reported vertigo in the present study did not correlate with vestibular test results. The lack of correlation between subjective vertigo and vestibular test outcome in the present study is not unique to CI rehabilitation but has been discussed for almost 30 years (66–68). This lack of correlation is also the central finding of the present study because vestibular disability, as reported by the patients, was not adequately captured by any of the vestibular diagnostic procedures in the current test battery. Interestingly, these results were found not only in patients with postoperative vertigo but also in the small group of patients who reported vertigo preoperatively and did not report vertigo postoperatively (n13, n16, and n24). The analysis of the questionnaire data indicated that one patient (n24) was suffering from vertigo of vestibular origin (group A), one patient (n16) had probably vestibular origin (group B), and in one patient (n13) no indication of vestibular origin was observed (group C). In patient n24, both vHIT and caloric measurements were outside the normal range pre- and post-operatively, supporting the strong suspicion of a vestibular origin of the reported vertigo based on the questionnaire evaluation. A similar result was seen in patient n16, in whom the caloric SPV was out of normal range only preoperatively, but SD was apparent at both test intervals and in whom, according to the questionnaire, a probable vestibular cause of vertigo could be assumed. This indicated that vestibular damage was present in these two patients already before surgery. Assuming that the test results were correct, reasons for the absence of vertigo could be a compensatory process that had occurred in the meantime or the impact of electrical stimulation postoperatively.

Multiple previous studies attempted to define the most reliable protocol for detecting vestibular impairment after CI. Abouzayd et al. (13) recently conducted a systematic review and, after a literature review, summarized the results of eight studies. Their meta-analyses calculated the sensitivity and specificity of the results of caloric irrigation, cVEMP, and vHIT using patient-reported symptoms as a reference. The pooled sensitivity of the caloric test was 21% (*n* = 6 studies), cVEMP 32% (*n* = 4), and vHIT 50% (*n* = 2). Despite certain limitations in interpretation (e.g., variable observation period after intervention, methodological differences), the poor sensitivity suggests that no single vestibular test is particularly sensitive to the relationship between subjective vertigo and vertigo diagnosis. We also hypothesize that a single individual vestibular test in isolation cannot provide sufficient information about the entire vestibular system or that the small group of patients was responsible for the limited validity of the test results.

### Low-Frequency Residual Hearing and Electrode Design

Hearing preservation after CI surgery is most likely equivalent to extensive cochlear structure preservation during surgery. The PTAlow results within electrode category group I confirmed the possibility of hearing preservation (Table 5); no case of complete loss of residual hearing was observed. In electrode category groups II and III, this was not consistently the case since four cases occurred with total loss of residual hearing in each group. The difference in PTAlow from pre- to postoperative was slightly larger in electrode category II than in electrode category I. This result is not unexpected since, in cases with preserved hearing, the extent of hearing loss caused by implantation is comparable. This fact is consistent with data available in the literature, as mentioned above. In individual category II cases and in electrode category III, this difference is not unexpected because of comparatively worse preoperative thresholds (e.g., PTAlow n21 = 101 dB) in combination with a floor effect limited by the maximum defined hearing loss (103 dB). This bias is responsible for the lack of significance of the PTAlow difference between groups I and III.

Although electrode category showed a correlation with the extent of hearing preservation, no correlation was found between electrode category and vertigo symptoms or the outcome of the various vestibular tests in this study.

### Insertion Angle and Electrode Design

Following the suggestion given by Helbig et al., two groups, depending on electrode insertion angle, were formed by subdividing the insertion angle above and below 430° (27). There was no correlation of insertion angle with the occurrence of self-reported vertigo or various parameters of the vertigo test battery. These results are consistent with those of Louza et al., who investigated a cohort of 41 cases (average insertion angle 464°). They reported no statistically significant correlation between insertion angle and the occurrence of vertigo or insertion angle and abnormal caloric irrigation test parameters (10). Nordfalk et al. (69) likewise investigated 39 cases (insertion angle, 405–708°) and reported no correlation between postoperative loss of vestibular function and insertion angle. Consistent with these observations, we did not observe a greater risk of vestibular impairment with deeper electrode insertion.

In order to minimize trauma after CI surgery, work has and will continue to improve the design of the electrode carriers. Due to the fact that category I or II electrodes are specifically or at least in principle suitable for hearing preservation, a reduced incidence of postoperative vertigo was expected for these electrode designs.

Thus, the results of the present study indicate no significant association between electrode category and onset of new vertigo. Similarly, in a prospective observational study by Krause et al. (70) comprising 36 patients implanted with pre-curved electrodes and 11 patients implanted with flexible straight electrodes, no significant difference related to electrode design was found in different parameters of postoperative vestibular diagnostics.

Different from these results, Frodlund et al. (71) reported a significant decrease in caloric response depending on electrode design, where in cases with straight electrodes (*n* = 15) and precurved electrodes (*n* = 13) SPV reduction of 23 and 7.6°/s was observed, respectively, whereas flexible electrodes showed no larger SPV decrease (0.1°/s, *n* = 15). Compared to the results of the present study (straight/precurved/flexible, −9/−7/+2.6°/s), a similar trend is obvious, however with smaller alterations. This might be related to the different number of cases (straight/precurved/flexible, *n* = 3/17/9) as well as the different test interval. The authors related the delayed onset of vertigo (1 month or later) to the occurrence of mechanical pressure generated by the electrode tip that might cause a lesion of the basilar membrane (71).

The prevalence of self-reported vertigo reported in the questionnaires after CI surgery was 44.8% (13/29 patients), and the incidence was 45.8% (10/24 patients), which is thus in agreement with the results of Krause et al., where an incidence of 45% was reported. In 10/13 cases (77%), new vertigo occurred within the first postoperative week. Thus, these results are similar to those of Krause et al., who reported an incidence of 80% in the same period. While the present study cohort did not report vertigo 3 months after surgery, Krause et al. mentioned one case (5%) with persistent vertigo. At the 6-month test interval, in the present study cohort, vertigo complaints appeared again in 3/13 cases (23%), whereas no vertigo case was reported by Krause et al. (1) at this interval.

Ito (63) categorized the patients with vertigo after cochlear implantation into different groups based on the temporal presence of their symptoms: the early type (vertigo within 2 weeks postoperatively), the prolonged type (persistent symptoms), and the delayed type (vertigo duration longer than 2 weeks). The author described that 58% belonged to the early type, 34% to the prolonged type, and 8% to the delayed type. In the present study, these percentages were 39% for the early type, 38% for the prolonged type, and 23% for the delayed type. In 2009, Hamann (72) described that, within the first 14 days after vestibular damage, there was a significant reduction to complete disappearance of vertigo. This observation had also been made previously by Black et al. (73), who described the likelihood for compensation over time. However, a postoperative increase of balance dysfunction is also possible. Thus, labyrinthitis could be a cause of late onset of cochlear implant-related vertigo (74). The study presented here does not provide evidence for the suspicion of labyrinthitis, as no signs of vestibular dysfunction were present in three cases with delayed-onset vertigo.

### Limitations of the Study

One major drawback of this study is the small number of participants (*n* = 29), which, in turn, reduced the size of the four subgroups. Although the aim was a before–after comparison after CI surgery, a control group could be considered an addition. It would be conceivable to include a group of patients who decide not to undergo cochlear implantation in the near future and are monitored for 6 months using the tests and questionnaires described above. Due to the small sample size of electrode group 1 (*n* = 5), the results of the statistical tests calculated by ANOVA and Tukey B are of limited validity. For this reason, the frequency of occurrence of postoperative deafness is probably the more appropriate factor to assess postoperative structural preservation.

## Conclusion

In the present study, the incidence of self-reported new onset of vertigo after cochlear implant provision was reported to be ~45%. As shown in a large number of previous studies, vestibular disorders are the most common complications after cochlear implantation. Consistent with this, we also found this complication to be frequent in the patient group studied here. The questionnaire evaluation confirmed new-onset vertigo after CI surgery in 72.7%, with symptoms suggestive of an otogenic etiology of vestibular dysfunction as outlined above. The symptoms indicated an otogenic etiology of vestibular dysfunction in 72.7% of all cases with new vertigo after CI surgery. As objective vestibular test results did not correlate with reported vertigo symptoms, an analysis of the origin of vestibular dysfunction after implantation was difficult. An effect of electrode design, in terms of insertion angle and shape, as an influencing factor for the occurrence of postoperative vertigo could not be confirmed. Further studies should clarify whether the lack of correlation between vestibular test results and reported vertigo is due to a lack of sensitivity of the currently applied methodologies, a central compensatory mechanism, or a multifactorial cause of vertigo.

## Data Availability Statement

The raw data supporting the conclusions of this article will be made available by the authors, without undue reservation.

## Ethics Statement

The studies involving human participants were reviewed and approved by Ethikkommission des Fachbereichs Medizin der Goethe-Universität Frankfurt am Main. The patients/participants provided their written informed consent to participate in this study.

## Author Contributions

CW, UB, and SH made substantial contributions to the conception, ethical approval of the work, and were responsible for the acquisition of patients and analysis as well as interpretation of data. ML and TS helped revising the work critically and added important intellectual content. All authors contributed to the article and approved the submitted version.

## Conflict of Interest

The authors declare that the research was conducted in the absence of any commercial or financial relationships that could be construed as a potential conflict of interest.
